# Inhibition of lipid and protein oxidation in raw ground pork by *Terminalia arjuna* fruit extract during refrigerated storage

**DOI:** 10.5713/ajas.17.0882

**Published:** 2018-07-26

**Authors:** Pranav Chauhan, Soubhagya Ranjan Pradhan, Annada Das, Pramod Kumar Nanda, Samiran Bandyopadhyay, Arun K. Das

**Affiliations:** 1 Division of Livestock Products Technology, ICAR-Indian Veterinary Research Institute, Bareilly -243 122, India; 2 Department of Livestock Products Technology, West Bengal University of Animal and Fishery Sciences, Kolkata-700 037, India; 3 Eastern Regional Station, ICAR-Indian Veterinary Research Institute, Kolkata-700 037, India

**Keywords:** Antioxidant, Ground Pork, Lipid Oxidation, Protein Oxidation, Sensory Attributes, *Terminalia arjuna* Fruit

## Abstract

**Objective:**

*Terminalia arjuna* plant, specially its leaves, bark, and roots, are widely used in traditional herbal medicine due to presence of bioactive components and being a rich source of natural antioxidants. But its fruit has not been used for any such purposes despite its potential to retard oxidation. Hence, the antioxidant potential of Arjuna fruit extract (AFE) in retarding lipid and protein oxidation of raw ground pork was evaluated during refrigerated storage for 9 days.

**Methods:**

The AFEs were prepared using different solvents *viz*. ethanol (EH), water, ethanol: water (60:40) and methanol:hot water (60:40). The AFEs were analysed for total phenolic content (TPC), 2, 2-diphenyl-1-picrylhydrazyl radical scavenging activity and reducing power. Water extract (WE) and ethanol-water extract (EH-WE) were selected and incorporated at 1.0% into freshly minced pork meat and compared with a synthetic antioxidant, in retarding lipid and protein oxidation during storage.

**Results:**

The TPC in AFEs using different solvents ranged from 11.04 to 16.53 mg gallic acid equivalents/g and extracts exhibited appreciable scavenging activity ranging from 50.02% to 58.62%. Arjuna extracts significantly (p<0.05) improved the colour score of meat samples by reducing the formation of metmyoglobin during storage. Both the AFEs (WE and EH-WE) significantly (p<0.05) lowered the thiobarbituric acid reactive substances value, peroxide formation and formation of protein carbonyls in raw pork than control sample during storage. Upon sensory evaluation of all samples, it was found that AFE treatment could prolong the storage period of meat samples, without influencing the colour and odour score, up to 6 days.

**Conclusion:**

AFEs used at 1% improved the oxidative stability, colour and odour score and prolonged the refrigerated shelf life of ground pork up 6 days. Therefore, AFE could be explored as an alternative natural antioxidant in retarding lipid and protein oxidation in meat products.

## INTRODUCTION

Lipid and protein oxidation are reported as major causes of quality deterioration in muscle foods. The oxidative changes are more intense in minced meat due to increased surface area and exposure to air during grinding and processing than intact meat. Proteins in muscle foods are prone to oxidation during processing and storage, which leads to loss of amino acids and solubility, changes in texture, formation of carbonyl, alterations in protein functionality and decreased sulfhydryl content [1]. These two factors not only brings in structural changes to protein but also lowers the flavour and colour scores, thereby declining the nutritive value of muscle food leading to an adverse effect on organoleptic attributes and consumer acceptability [2,3]. Although reports on the effects of lipid oxidation on muscle foods during refrigerated storage are available, but studies on protein oxidation in fresh and processed meat products are scanty [2]. To overcome the problem, natural antioxidants from various plant extracts are being used to control protein and lipid oxidation in muscle foods. Over the years, with increased awareness level among the consumers, the demand for natural antioxidants has increased steadily over synthetic antioxidants in the processed food industry. Recent reports on inhibitory effect of various plant extracts, such as phenolics from pomegranate peel [2], litchi pericarp [4], rapeseed and pine bark [3] on protein and/or lipid oxidation in muscle food system are testimony to this. However, till date, hardly any study has been conducted to investigate the efficacy of *Terminalia arjuna* (*T. arjuna*; Combretaceae) fruit extract, in retarding lipid and protein oxidation in meat food system, although its leaves, bark, and roots are extensively used as herbal medicine in both traditional Ayurvedic and Yunani systems [5]. Keeping in view the medicinal importance and antioxidant activity of this plant, the present study was carried out to investigate the antioxidant and antimicrobial properties of *T. arjuna* fruits, and to explore the antioxidant efficacy of fruit extracts in retarding lipid and protein oxidation of raw ground pork during refrigerated storage.

## MATERIAL AND METHODS

### Preparation of *Terminalia arjuna* fruits extract

Fully matured *T. arjuna* fruits were taken for this purpose. After cleaning extraneous dirt, the outer covering was scrapped. The fruits were dried in a hot air oven at 45°C±2°C for 8 to 10 h and ground in a grinder (Kenstar, Mumbai, India), till fine powder was obtained. Arjuna fruit extract (AFE) were prepared using different solvents viz. ethanol (EH), water (WE), ethanol:water (EH-WE, 60:40) and methanol:water (MH-WE, 60:40). For each formulation, 10 g powder was accurately weighed into a flask to which 100 mL solvent was added. The flasks were kept overnight at room temperature (27°C±1°C) with constant shaking (400 rpm for 8 h), vortexed at high speed for 10 min. The content was finally centrifuged at 5,000 rpm for 10 min and passed through filter paper to get AFE.

### Analysis of total phenolic content

The total phenolics content (TPC) of AFE was estimated following the protocol of Singleton and Rossi [6]. Briefly, 100 μL of AFE was mixed with 0.75 mL of Folin-Ciocalteu reagent and the volume was made up to 8.5 mL with distilled water (DW) and kept for 5 min at room temperature. To this, sodium bicarbonate (0.75 mL of 6%) was added and the content was incubated in dark (at room temperature) for 90 min. After the incubation period (90 min), absorbance was measured against a blank at 765 nm (Eppendorf BioSpectrophotometer, Hamburg, Germany). A standard curve was plotted using different concentrations of gallic acid, and the amount of total phenolic was calculated as gallic acid equivalents (GAE) in mg/g of fruit powder.

### Radical scavenging activity using 2, 2-diphenyl-1-picrylhydrazyl assay

Different concentrations (0.2, 0.4, 0.6, 0.8, 1.0 mL) of AFE were taken in test tubes and the volume was made up to 4 mL with DW [7]. To this, 1 mL of 1 mM 2, 2-diphenyl-1-picrylhydrazyl (DPPH) methanolic solution was added. All the test tubes were shaken well and allowed to stand for 30 min at room temperature. A control sample was prepared by mixing 1 mL DPPH solution with 4 mL of DW and absorbance was immediately taken at 517 nm (DW used as blank). Free radical scavenging activity (FRSA) was calculated using the following formula:

$$\text{FRSA }(\%)=\frac{{\text{Absorbance}}_{\text{Control}}-{\text{Absorbance}}_{\text{Sample}}}{{\text{Absorbance}}_{\text{Control}}}\times 100$$

### Ferric reducing antioxidant power assay

The reducing power of the AFE was determined according to the method of Oyaizu [8]. Briefly, different concentrations of AFEs were mixed with 2.5 mL of phosphate buffer (0.2 M, pH 6.6) and 2.5 mL of 1% (w/v) potassium ferricyanide and incubated for 20 min at 50°C [8]. This was followed by addition of 2.5 mL of 10% trichloroacetic acid (TCA) and centrifugation at 1,500 rpm for 10 min. About 2.5 mL of supernatant was mixed with equal volume of DW and 0.5 mL of ferric chloride (0.1% w/v), and the absorbance was measured at 700 nm. An increase in the absorbance of the reaction mixture indicated the reducing power of the sample. The extracts showing maximum yield in terms of TPC; DPPH radicals scavenging activity and ferric reducing antioxidant power (FRAP) assay were selected for the study purpose.

### Preparation of raw pork meat samples

Meat was collected from Ghungroo pig breed and minced (through a 6 mm grinding plate followed by 4 mm plate) in a meat mincer. Based on the TPC and radical scavenging activity, Arjuna extracts (WE and EH-WE) were selected and incorporated at 1% into freshly minced pork meat. Meat samples were divided into four different batches. The control (C) sample (meat without any extract) contained 1% DW, while treatment groups (T1 and T2) contained WE and EH-WE at 1%, respectively. The third treatment group (T3) included a synthetic antioxidant, butylated hydroxyl toluene (BHT, 100 ppm). Each treatment group was blended for 2 min, subdivided into five groups and then, aerobically packaged in low-density polyethylene bags. The samples were stored at refrigerated temperature (4°C±1°C) and analysed at three day interval for various parameters (pH, colour and odour score, peroxide, thiobarbituric acid reacting substances [TBARS], total carbonyl contents and total plate count) up to 9 days.

### Metmyoglobin content (%)

About 3 g of ground pork meat was blended with 30 mL of 0.04 M chilled phosphate buffer (pH 6.8). The meat sample was homogenised with the help of pestle and mortar for 20 s and kept for one hour at refrigerated temperature (4°C). Then, the samples were centrifuged at 8,000 rpm for 10 min in a refrigerated centrifuge (Eltek MP- 400-R Eltek India, Delhi, India) at 4°C. The supernatant collected was filtered through a Whatman filter paper No. 42. The optical density was measured in a UV-VIS spectrophotometer (Eppendorf BioSpectrophotometer, Germany) at 525, 572, and 700 nm and the percentage metmyoglobin (MMb) was calculated using the formula of Koniecko [9].

$$\text{MMb }(\%)=\frac{1.395-({\text{OD}}_{572}-{\text{OD}}_{700})}{\left({\text{OD}}_{525}-{\text{OD}}_{700}\right)}\times 100$$

### Peroxide values

Peroxide value (PV) of meat sample was determined as per the procedure described by Koniecko [9] with slight modifications. For PV, meat sample (5 g) was blended with anhydrous sodium sulphate (5 g) and 30 mL chloroform. The mixture was filtered through Whatman filter paper No. 1. To 25 mL aliquot of the filtered chloroform extract; 30 mL of glacial acetic acid and 2 mL of saturated potassium iodide solution were mixed. After 2 min, 100 mL of DW and 2 mL of fresh 1% soluble starch, Amylum (Himedia, Mumbai, India) solution were added to the content and titrated immediately against 0.1 N sodium thiosulphate till the end point was reached (non-aqueous layer turned to colourless). The PV was expressed in mEq/kg of the sample.

### Thiobarbituric acid reacting substances values

Lipid oxidation was performed by measuring TBARS values following the method of Witte et al [10] with slight modifications. Briefly, 10 g of sample from each treatment group was triturated with 25 mL of pre-cooled 20% TCA for 2 min. The content was then quantitatively transferred into a beaker by rinsing with 25 mL of chilled DW, mixed and filtered. Three millilitres of TCA extract (filtrate) was mixed with 3 mL of thiobarbituric acid (TBA) reagent (5 mM) in test tube and cooled in a running tap water after boiling in a water bath at 70°C for 35 minutes. A blank sample was made by mixing 3 mL of 10% TCA and 3 mL of 5 mM TBA reagent. The absorbance was measured at a fixed wavelength of 532 using a UV-VIS spectrophotometer. The TBA value was calculated as mg malonaldehyde per kg of the sample by multiplying the absorbance value with a factor of 5.2.

### Protein oxidation (total carbonyl content) of raw pork meat

Estimation of total carbonyl content was carried out according to the method outlined by Vuorela et al [3] through two different measurements such as quantification of (a) carbonyls and (b) protein. Minced pork meat (1 g) was homogenised with 10 mL of 0.15 M KCl in an Ultraturrax homogenizer for 1 min. To each 100 μL of homogenate, 1 mL of 10% TCA was added. The contents were centrifuged at 5,000 rpm for 5 min, and the supernatant was removed. For sample (a) 1 mL of 2 M HCl with 0.2% DNPH and for sample (b) 1 mL of 2 M HCl was added. After 1 h of incubation (shaken every 20 min), 1 mL of 10% TCA was added. The contents were vortexed and centrifuged at 5,000 rpm for 5 min. The supernatant was removed carefully with a Pasteur pipette without disturbing the pellet. The pellet was washed with 1 mL of ethanol/ethyl acetate (1:1), shaken, and centrifuged at 10,000 rpm for 5 min. This procedure was repeated at least two times. The pellet so obtained was completely dried with nitrogen, dissolved in 1.5 mL of 20 mM sodium phosphate buffer with 6 M guanidine hydrochloride (pH 6.5), shaken and centrifuged at 5,000 rpm for 2 min. Carbonyls (sample a) and protein concentration (sample b) were measured spectrophotometrically at 370 nm and 280 nm, respectively. Protein quantification was determined using a standard curve made from bovine serum albumin [3]. Concentration of carbonyls was calculated based on the equation mentioned below where 21.0 mM^−1^ cm^−1^ is the molar extinction coefficient of carbonyls.

$$\text{Carbonyls}=\frac{{\text{Abs}}_{370\text{nm}}}{21.0\;{\text{mM}}^{-1}\;{\text{cm}}^{-1}}\times 1,000$$

### Total plate count

The total plate count of samples from different treatment groups was determined at different storage intervals by using pour plate method [11]. A 10 g of meat sample was homogenised in 90 mL of sterile peptone water (0.1%). Appropriate serial dilutions were prepared in 0.1% sterile peptone water and duplicate plated with plate count agar, incubated at 37°C for 48 h. Microbial colonies from the plates were counted and expressed as log_10_ colony-forming unit (CFU)/g.

### Colour and odour score of raw ground meat

Colour and odour scores were evaluated by a panel of seven judges consisting of faculty and postgraduate students. Prior to sensory evaluation, the panellists were trained using 14 extra samples for 3 sessions before they started on real samples on the days of analysis. Samples were placed in covered cups coded with random 3-digit numbers and allowed to keep at room temperature prior to evaluation. In the case of colour, a 5-point descriptive scale was used where 1 = pale pink, 2 = pink, 3 = pinkish red, 4 = bright red, and 5 = reddish brown. Similarly, for sensory odour score, a 5 point scale was used where 1, very unpleasant; 2, moderately unpleasant; 3, moderately pleasant; 4, pleasant; and 5, very pleasant [11].

### Statistical analysis

The present study was replicated three times and in each time, measurement of all parameters was done in duplicate. The data analysis was carried out using SPSS software (version 20.0; IBM, Armonk, NY, USA). Treatment (control, T1, T2, and T3) and storage period (0, 3, 6, and 9 days) were considered as factors. This storage study was also replicated three times and carried out in 4×4 factorial design according to complete randomized design. The data were subjected to analysis of variance and Duncan’s multiple range test was used for comparing the means to find out the effect of treatment and storage period on various parameters. The values were presented as mean along with standard error (mean±standard error) and significance level was identified at the 95% confidence level (p<0.05).

## RESULTS AND DISCUSSION

### Total phenolic content

The amount of total phenolic content (TPC) extracted from Arjuna fruits using different solvents varied widely and is presented in Table 1. Among all the solvent extracts tested; EH-WE had the highest TPC (16.53 mg GAE/g powder) followed by WE (13.42 mg GAE/g), EHE (11.85 mg GAE/g), and MH-WE (11.04 mg GAE/g). Such differences in the effectiveness of solvents in extracting phenolics might be attributed to affinity of the endogenous compounds towards particular solvent, chemical composition of fruits, nature of soil and agro-climatic conditions of the area from which fruits are obtained [12]. As reports on TPC and antioxidant activity of AFE are very scanty, results of this study were correlated with the findings of *T. arjuna* bark or leaves extract. Sultana et al [13] reported similar range of TPC in aqueous ethanolic extract using *T. arjuna* bark (12.8 5mg GAE/g of DW). Bajpai et al [14] reported that the leaves, bark and fruits of *T. arjuna*, *T. bellerica*, *T. chebula*, and *T. muelleri* had high TPC (72.0 to 167.2 mg/g GAE) in relation to high antioxidant activity (69.6% to 90.6%). Such variations in the amount of TPC in different plant components could be due to difference in maturity conditions of fruit at harvest, growing conditions, soil conditions, and post-harvest storage treatment etc. [15].

### DPPH radical scavenging activity of Arjuna fruit extract

In the present study, AFE using different solvents showed a varied potential of antioxidant capacities in term of inhibitory concentration (IC_50_) (μg/mL) ranging from 10.37 to 12.98 μg/mL and is depicted in Table 1. The IC_50_ value of aqueous ethanolic fruit extract was lowest (10.37 μg/mL) showing the highest per cent inhibition among all the extracts whereas highest IC_50_ (12.98 μg/mL) showing the lowest per cent inhibition of aqueous methanolic fruit extract. It is well-known facts that lower the IC_50_ values of extract, higher will be the inhibition of DPPH free radicals and vice versa. Our findings can be correlated with IC values of other medicinal plant extracts [13]. In a similar study, the potential of antioxidant properties of arjuna leave and stem bark extracts in terms of IC_50_ (2.71 to 7.68 μg/mL) has also been reported [5]. The fruit extracts using different solvents exhibited appreciable scavenging activity ranging from 50.02% to 58.62%. Significantly (p<0.05) higher and lower scavenging activity was observed in Arjuna fruit extracted with ethanol-water (EH-WE) and methanol-water (MHW), respectively. Sultana et al [13] reported similar scavenging activity, ranging from 49.0% to 87.0%, using extracts from barks of *Azadirachta indica*, *T. arjuna*, *Acacia nilotica*, and *Eugenia jambolana* Lam. tree. Substantial DPPH radical scavenging capacity of the fruit extracts could be explained by the presence of phenolic components in these solvent extracts.

### Ferric reducing antioxidant power of Arjuna fruit extracts

Reducing power is a measure to directly correlate the amount of phenolic compounds in extracts or samples. The reducing power of the AFE, analysed at 0.5 to 2.0 mg/mL, increased as the concentration increased. The reducing power of AFE (2.0 mg/mL) ranged from 0.748±0.019 to 1.33±0.025, with water and ethanol extracts exhibiting the highest and lowest reducing power, respectively. Our results are comparable with the findings of Sultana et al [13] who also reported reducing potential of *T. arjuna* bark extracts using different solvents.

### pH and microbial status of ground pork meat

The effect of various treatments (WE, EH-WE, and BHT) on pH values of meat samples is presented in Table 2 and compared with control. At the beginning of storage (0 day), pH value was not significantly (p>0.05) different between control and the treatments. However, on day 3, the pH was significantly (p<0.05) higher in the control sample (C) compared to treated sample (T1, T2, and T3) and the trend was similar up to 9th day of storage period (Table 2). The increase in pH values of control samples on 9th day of storage study could be due to accumulation of ammonia and the products of amino acid released during protein degradation and utilisation of amino acids by bacteria. Biswas et al [16] also reported similar pH values of pork meat sample treated with curry leaf and mint extract stored for a period of 12 days.

Table 1 shows the effect of different treatments on microbial load of pork meat compared to control samples during 9 day refrigerated storage study. Result shows that aerobic plate count (APC) ranged from 3.03 to 3.40 log_10_ CFU/g meat on 0 day and the count reached to 5.15 to 6.67 log_10_ CFU/g of meat sample on 9th day. However, APC of treated ground pork meat samples (T1 and T2) was significantly (p<0.05) lower than control. This might be due to the richness of treated samples, notably T1 and T2, in polyphenolic compounds having antimicrobial effect along with antioxidant property. In fact, polyphenols are well documented to have microbiocidal activities against a huge number of pathogenic bacteria. Ramya et al [17] reported that aqueous extracts of *T. arjuna* leaves and fruits were active against Gram negative compared to Gram positive organisms. Vaithiyanathan et al [18] reported that precipitable phenolics and condensed tannins of pomegranate fruit juice phenolics, when used in chicken meat have antimicrobial property. From this, it can be concluded that presence of phenolics of AFE in treated samples could have inhibited the microbial growth in treated samples, either through protein binding or enzyme inhibition.

### Metmyoglobin content of ground pork meat

The effect of different treatments on metmyoglobin (MMb) percentage of raw ground pork was studied and compared with control over 9 days of refrigerated storage and is presented in Table 2. At the beginning of the storage, the MMb (33.91% to 37.13%) values were not significantly different between control and treatments groups. But ground pork meat treated with either WE or EHWE had significantly (p<0.05) lower percentage of MMb values (48.48% and 47.27%, respectively) even on 9th day of storage compared to control (67.23%) and BHT treated (T3) sample (49.58%). Our findings are in agreement with study conducted by Jo et al [19] who successfully used green tea ethanolic extract to delay the loss of redness in raw pork patties during storage. Such delay in loss of redness in raw pork meat treated with AFE could be due to lower lipid oxidation and microbial growth due to the presence of good amount of TPC and antioxidant activity. On the other hand, the increase in MMb value in untreated samples could be attributed to the increased oxidation of ferric iron into the ferrous state and increased rate of lipid oxidation converting oxymyoglobin to metmyoglobin [20].

### Thiobarbituric acid reactive substances of ground pork meat

**Figure 1:**
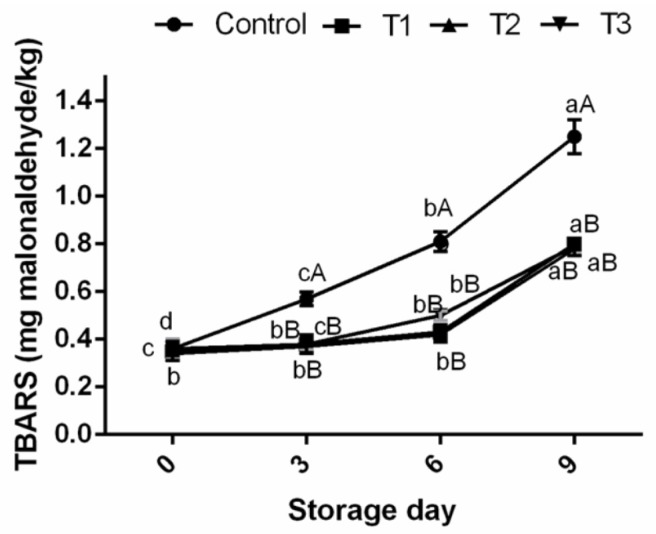
Effect of Arjuna fruit extract (WE and EH-WE) and BHT on TBARS of ground pork meat during refrigeration storage. WE, water extract; EH-WE, ethanol-water extract; BHT, butylated hydroxyl toluene; TBARS, thiobarbituric acid reacting substances. Control, no additive; T1, WE; T2, EH-WE (60:40); T3, 100 ppm BHT; n = 6. ^a–c^ Means with different superscripts are significantly different (p<0.05) among storage periods. ^A–D^ Means with different superscripts are significantly different (p<0.05) among treatments.

TBARS assay is one of the most widely used methods for measuring secondary oxidation products which are known as the cause of oxidative rancidity. In this study, lipid oxidation, expressed as TBARS, in control ground pork increased rapidly during refrigerated storage, reaching 1.25 mg/kg compared to treated samples (≤0.8 mg malondialdehyde/kg sample) on 9th day of storage study (Figure 1). It is quite evident that the TBARS production was significantly inhibited (p<0.05) in ground pork in treated groups (T1, T2, and T3) and decreased by 56.25%, 60.25%, and 58.23%, respectively, compared to the control over 9 days storage period. The efficacy of AFE was comparable with that of BHT and incorporation of AF-WE and AF-EHWE at 1% in ground pork samples reduced TBARS values similar to that of BHT (100 ppm) indicating their protective effect against lipid oxidation. Many researchers have also documented the effects of fruit extracts as inhibitors of lipid oxidation in muscle foods. Pomegranate fruit peel extracts were found to reduce TBARS values of beef meat ball at refrigerated storage temperature compared to control sample [2]. Extracts from litchi fruit pericarp protected lipid against oxidation in sheep meat nuggets stored for 12 days at 4°C [4]. The present study clearly shows that AFE could be an effective source of natural antioxidant in preventing ground pork against lipid oxidation at refrigerated storage.

### Peroxide values of ground pork meat

As the primary products of lipid oxidation are hydroperoxides, it seems reasonable to determine the concentration of peroxide in order to estimate the extent of initial oxidation in meat samples. The results presented in Table 3 indicate that the initial PV (3.39 mEq/kg) of control sample increased to 5.23 mEq/kg after 9 days of storage, which was significantly higher (p<0.05) compared to treated samples (T1, T2, and T3). Similarly, when ground pork treated with mustard leaf kimchi ethanolic extracts and ascorbic acid found to have significantly lower PV during storage [21]. These results suggested that the control samples underwent a noticeable lipid oxidation during the first 6 days of refrigerated storage and reached to maximum PV at the end of the primary auto-oxidation. After 6 days of storage, the hydroperoxides formed might have gone through the decomposition to form secondary lipid oxidation products [22]. Although oxidation in the control was more intense compared to the treated samples; a decline was observed on day 6. This indicates that after the induction period, the decomposition rate of the hydroperoxides was faster than the production rate. But in case of AFE and BHT treated samples, an increase in hydroperoxide formation was observed even on 9th day of storage compared to control. This indicated that progression of initial oxidation step and the degradation of the peroxides formed were very slow in AFE treated samples retarding further progression of oxidation. The results are in line with earlier report on effects of grape seed extract on PVs in refrigerated storage study of pork frankfurters [23].

### Protein oxidation of ground pork meat

**Figure 2:**
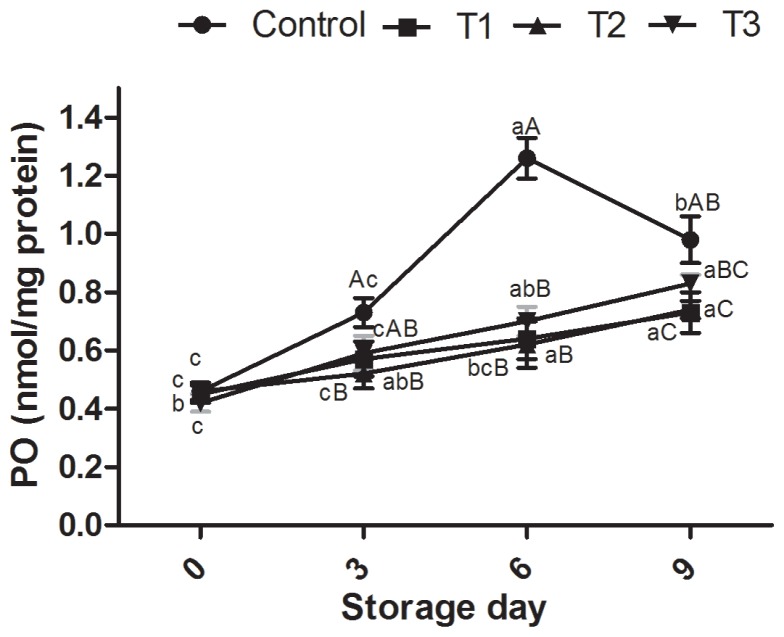
Effect of Arjuna fruit extract (WE and EH-WE) and BHT on protein oxidation (PO) of ground pork meat during refrigerated storage. WE, water extract; EH-WE, ethanol-water extract; BHT, butylated hydroxyl toluene. Control, no additive; T1, WE; T2, EH-WE (60:40); T3, 100 ppm BHT; n = 6. ^a–c^ Means with different superscripts are significantly different (p<0.05) among storage periods. ^A–D^ Means with different superscripts are significantly different (p<0.05) among treatments.

The mean carbonyl content in control was 0.857 nmole/mg protein, while it was 0.59, 0.58, and 0.64 nmole/mg protein in raw ground pork with AF-WE, AF-EHWE at 1% and BHT (100 ppm), respectively. Increase of carbonyl level during storage in all the samples indicated that oxidative reaction occurred during storage (Figure 2). Initial carbonyl concentrations of samples (C, T1, T2, and T3) were 0.46, 0.45, 0.46, and 0.42 nmole/mg protein, respectively. The final carbonyl level of control samples was 0.98 nmole/mg protein which was higher than the antioxidant treated (T1, T2, and T3) samples with final carbonyl concentrations of 0.73, 0.74, and 0.83 nmole/mg protein, respectively. Control samples at all sampling days had significantly higher amounts of protein carbonyls than the antioxidant treated samples. These results suggest that samples treated with AFE had significant effect in controlling final carbonyl concentrations. Different researchers have also reported inhibitory effects of plant extracts such as strawberry tree, common hawthorn, dog rose and elm-leaf blackberry in decreasing carbonyl formation in burger patties [24], pork patties [3] and beef meatball [2] during storage study. It is well known that protein carbonyls are formed mostly by the interaction between the proteins and the aldehydes formed as a result of lipid oxidation [25] and the formation of protein carbonyls can cause protein degradation, fragmentation or aggregation [26]. In this study, control raw ground pork meat samples had significantly higher amounts of protein carbonyls than the antioxidant treated samples. The maximum concentration of carbonyl, approximately three-fold the initial level, was recorded in control sample on day 6. Further, increase in carbonyl content in control sample was higher than the increase in TBARS on day 6 indicating that protein oxidation occurred more rapidly than lipid oxidation. These results are in agreement with those previously studied in muscle food system [2,24].

### Colour and odour score of ground pork meat

Colour is an important sensory attribute, which determines the products’ acceptability rate. During initial storage study, no difference was observed in colour scores between, control and different treatments groups (Table 2). On 6th day, control sample had significantly (p<0.05) lower colour score, while no significant difference was observed between BHT and AFE treated samples. Furthermore, incorporation of WE and EHWE did not negatively affect colour of ground pork. In a similar study, Devatkal and Naveena [27] did not observe any negative effect of pomegranate peel extract on sensory properties of raw or cooked meat products.

It is an established fact that the colour of meat and meat products is influenced by the percentage of metmyoglobin in muscle [28]. In fact, the myoglobin is transformed into oxymyoglobin (light pink colour), which results in brighter red meat, and upon oxidization is converted into metmyoglobin during storage. The decrease in redness score in control sample over the storage time in the present study could be associated with the oxidation of myoglobin to metmyoglobin during refrigerated storage. However, AFE could improve the colour score in raw ground pork during refrigerated storage because of its antioxidant properties preventing the oxidation of oxymyoglobin. Zhang et al [29] reported that incorporation of mulberry leaves extract could improve a* value (redness) in raw ground beef during refrigerated storage. Kim et al [30] demonstrated that adding soy sauce could retard the decrease of a* value (redness) and reduce the production of metmyoglobin in raw beef patties during cold storage.

During oxidative degradation of unsaturated fatty acids, present in the meat systems, undesirable flavour and odour occur due to the formation of aldehydes, ketones and alcohols [31]. In this study, rancid odour development increased (the scores decreased) for all treatments as storage time increased, which was indicative of lipid oxidation of ground pork. The AFE and BHT treated samples were fairly stable up to 6 days and no significant differences (p<0.05) in the intensities of their off-odour characteristics were observed, while rancid odour development was quite apparent in the control sample. This could be due to decrease in oxidation because of the antioxidative properties of AFE and BHT thereby lowering the off-odour or rancid odour development in treated samples. Furthermore, the differences in rancidity development between control and treated pork meat samples were in similar line to TBARS values recorded on day 6. Our results are in agreement with reports of Jayathilakan et al [32] who observed that clove and cinnamon extracts were very effective in decreasing the worm-over flavour development and the hexanal contents in fresh meat samples during storage. Das et al [11] also reported that natural antioxidant such as curry leave powder reduced off-odour intensities in fresh goat meat. Thus, on the basis of sensory and oxidative studies, it can be concluded that shelf-life of ground pork meat could be extended up to 6th day in AFE treated samples.

## CONCLUSION

From this study, it can be concluded that Arujan fruit (WE and EH-WE) possesses higher phenolic content with promising free radical scavenging activity and reducing power. Incorporation of AFE can significantly retard TBARS, peroxide formation in ground pork. Phenolic compounds present in AFE were also found to possess promising protein oxidation retarding capacity. Incorporation of AFE at 1% can significantly lower the rancid odour development of ground pork during refrigerated storage without having any negative impact even on colour. In pork and pork products, it is more relevant owing to their sensitivity to protein oxidation and rancidity. Therefore, incorporation of AFE at 1% in muscle foods not only helps in retarding lipid and protein oxidation thereby enhancing the shelf life, quality and safety of muscle foods, but also fulfils the consumers’ perception on use of natural antioxidants in processed food as well.

## Figures and Tables

**Table 1:** Total phenolic contents and DPPH radical scavenging activity of Arjuna fruit extract1)

Solvents	TPC (mg GAE/g)	DPPH IC_50_ (μg/mL)
Water extract	13.42±0.77^ab^	11.42±0.45^ab^
Ethanol extract	11.85±1.14^b^	12.86±0.32^a^
Ethanol:water extract (60:40)	16.53±1.43^a^	10.37±0.28^b^
Methanol:water extract (60:40)	11.04±0.53^b^	12.98±0.36^a^

DPPH, 2, 2-diphenyl-1-picrylhydrazyl; TPC; total phenolic contents, GAE; gallic acid equivalents; IC_50_, inhibitory concentration.

1) n = 6.

ab Values (mean±standard error) bearing different superscript in a column differ significantly (p<0.05).

**Table 2:** Effect of Arjuna fruit extracts (WE and EH-WE) and BHT on pH, aerobic plate count, metmyoglobin and colour score of pork meat during refrigeration storage

Treatment1)	Storage days			

	0	3	6	9
	pH			
Control	6.05±0.01^d^	6.19±0.04^c^^A^	6.30±0.04^b^^A^	6.59±0.01^a^^A^
T1	6.07±0.01^b^	6.11±0.02^ab^^B^	6.14±0.02^ab^^B^	6.15±0.06^a^^B^
T2	6.06±0.01^b^	6.09±0.03^ab^^B^	6.12±0.01^ab^^B^	6.14±0.03^a^^B^
T3	6.03±0.04^b^	6.08±0.03^ab^^B^	6.13±0.02^ab^^B^	6.17±0.02^a^^B^
	Aerobic plate count (log_10_ CFU/g)			
Control	3.39±0.39^d^^A^	4.25±0.11^c^^A^	5.48±0.14^b^^A^	6.67±0.12^a^^A^
T1	3.11±0.10^d^^AB^	3.63±0.13^c^^B^	4.53±0.15^b^^B^	5.20±0.05^a^^B^
T2	3.03±0.06^d^^B^	3.59±0.11^c^^B^	4.67±0.14^b^^B^	5.15±0.11^a^^B^
T3	3.40±0.08^d^^A^	4.21±0.07^c^^A^	5.42±0.16^b^^A^	6.62±0.20^a^^A^
	Metmyoglobin (%)			
Control	37.13±2.27^d^	49.36±1.35^c^^A^	58.40±1.79^b^^A^	67.23±1.32^a^^A^
T1	34.17±1.39^d^	40.69±0.48^c^^B^	44.85±0.84^b^^B^	48.48±1.42^a^^B^
T2	33.47±1.08^d^	39.86±0.73^c^^B^	43.24±0.73^b^^B^	47.27±1.14^a^^B^
T3	33.91±1.98^d^	40.62±0.69^c^^B^	45.17±0.79^b^^B^	49.58±1.10^a^^B^
	Colour score			
Control	4.63±0.16^a^	3.95±0.12^b^^B^	2.90±0.12^c^^B^	1.82±0.16^d^^B^
T1	4.65±0.07^a^	4.58±0.12^a^^A^	4.10±0.10^b^^A^	3.05±0.15^c^^A^
T2	4.67±0.13^a^	4.60±0.14^a^^A^	4.08±0.12^b^^A^	3.07±0.14^b^^A^
T3	4.67±0.11^a^	4.55±0.07^a^^A^	4.09±0.08^b^^A^	3.09±0.15^c^^A^

WE, water extract; EH-WE, ethanol-water extract; BHT, butylated hydroxyl toluene; CFU, colony-forming unit; SE, standard error.

1) Control, no additive; T1, WE; T2, EH-WE (60:40); T3, 100 ppm BHT; n = 6.

a–c Mean±SE with different small letter superscripts on the same row are significantly different (p<0.05).

A–D Mean±SE with different capital letter superscripts on the same column are significantly different (p<0.05).

**Table 3:** Effect of Arjuna fruit extracts (WE and EH-WE) and BHT on peroxide value and odour score of pork meat during refrigeration storage

Treatment1)	Storage days			

	0	3	6	9
	Peroxide value (mEq/kg)			
Control	3.39±0.19^d^	4.54±0.13^c^^A^	6.17±0.09^b^^A^	5.23±0.19^a^^A^
T1	3.18±0.16^c^	3.73±0.21^bc^^B^	4.10±0.17^b^^B^	4.91±0.07^a^^B^
T2	3.19±0.14^c^	3.69±0.26^bc^^B^	4.04±0.19^b^^B^	4.88±0.22^a^^B^
T3	3.16±0.27^c^	3.71±0.25^bc^^B^	4.14±0.15^b^^B^	5.04±0.12^a^^B^
	Odour score			
Control	4.93±0.03^a^	4.57±0.14^a^^B^	2.82±0.12^b^^B^	1.90±0.15^c^^B^
T1	4.95±0.03^a^	4.87±0.03^a^^A^	4.10±0.06^b^^A^	2.85±0.13^c^^A^
T2	4.95±0.02^a^	4.92±0.04^a^^A^	4.18±0.10^b^^A^	2.97±0.12^c^^A^
T3	4.93±0.03^a^	4.88±0.05^A^	4.07±0.07^b^^A^	2.73±0.10^c^^A^

WE, water extract; EH-WE, ethanol-water extract; BHT, butylated hydroxyl toluene; SE, standard error.

1) Control, no additive; T1, WE of *Arjuna* fruit; T2, EH-WE (60:40) of *Arjuna* fruit; T3, 100 ppm BHT; n = 6.

a–c Mean±SE with different small letter superscripts on the same row are significantly different (p<0.05).

A–D Mean±SE with different capital letter superscripts on the same column are significantly different (p<0.05).
